# Supplementary material for: Evidence supported by Mendelian randomization: impact on inflammatory factors in knee osteoarthritis
Source: Front Med (Lausanne). 2024 May 28;11:1382836. doi: 10.3389/fmed.2024.1382836 (PMC11165061; doi:10.3389/fmed.2024.1382836)
Supplement: Supplementary file 1 [file Data_Sheet_1.ZIP › data/GCST90274775_5e6/775.forest.pdf]

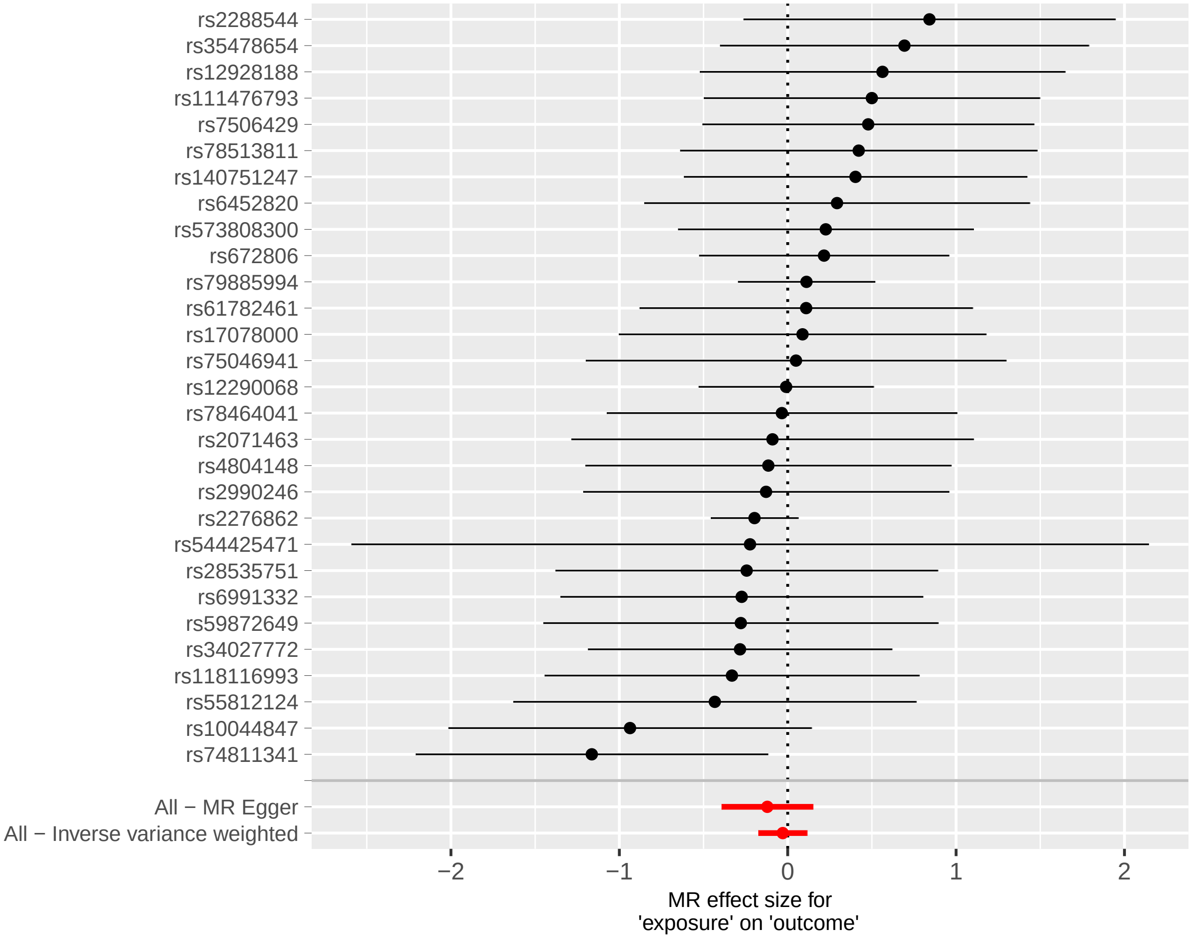
rs2288544
rs35478654
rs12928188
rs111476793
rs7506429
rs78513811
rs140751247
rs6452820
rs573808300
rs672806
rs79885994
rs61782461
rs17078000
rs75046941
rs12290068
rs78464041
rs2071463
rs4804148
rs2990246
rs2276862
rs544425471
rs28535751
rs6991332
rs59872649
rs34027772
rs118116993
rs55812124
rs10044847
rs74811341
All − MR Egger
All − Inverse variance weighted
−2
−1
0
1
2
MR effect size for
'exposure' on 'outcome'
